# Relatively frequent switching of transcription start sites during cerebellar development

**DOI:** 10.1186/s12864-017-3834-z

**Published:** 2017-06-13

**Authors:** Peter Zhang, Emmanuel Dimont, Thomas Ha, Douglas J. Swanson, the FANTOM Consortium, Winston Hide, Dan Goldowitz

**Affiliations:** 1 Centre for Molecular Medicine and Therapeutics, Child and Family Research Institute, Department of Medical Genetics, University of British Columbia, 950 West 28th Avenue, Vancouver, BC V5Z 4H4 Canada; 2 Department of Biostatistics, Harvard School of Public Health, 655 Huntington Ave, Boston, MA 02115 USA; 3 Harvard Stem Cell Institute, 1350 Massachusetts Ave, Cambridge, MA 02138 USA; 4 Department of Neuroscience, Sheffield Institute of Translational Neuroscience, University of Sheffield, Room B37 385a Glossop Road, Sheffield, South Yorkshire S10 2HQ UK

**Keywords:** Cerebellum, Developmental biology, Promoter, Promoter switching, HeliScopeCAGE, Alternative promoters, Alternative splicing, Transcription start site

## Abstract

**Background:**

Alternative transcription start site (TSS) usage plays important roles in transcriptional control of mammalian gene expression. The growing interest in alternative TSSs and their role in genome diversification spawned many single-gene studies on differential usages of tissue-specific or temporal-specific alternative TSSs. However, exploration of the switching usage of alternative TSS usage on a genomic level, especially in the central nervous system, is largely lacking.

**Results:**

In this study, We have prepared a unique set of time-course data for the developing cerebellum, as part of the FANTOM5 consortium (http://fantom.gsc.riken.jp/5/) that uses their innovative capturing of 5′ ends of all transcripts followed by Helicos next generation sequencing. We analyzed the usage of all transcription start sites (TSSs) at each time point during cerebellar development that provided information on multiple RNA isoforms that emerged from the same gene. We developed a mathematical method that systematically compares the expression of different TSSs of a gene to identify temporal crossover and non-crossover switching events. We identified 48,489 novel TSS switching events in 5433 genes during cerebellar development. This includes 9767 crossover TSS switching events in 1511 genes, where the dominant TSS shifts over time.

**Conclusions:**

We observed a relatively high prevalence of TSS switching in cerebellar development where the resulting temporally-specific gene transcripts and protein products can play important regulatory and functional roles.

## Background

Alternative splicing can provide a large reservoir of transcriptional variants from the ~22,000 genes identified by the Human Genome Project [1]. The production of different isoforms due to the usage of alternative transcription start sites (TSSs), which was once considered as uncommon, has now been found in the majority of human genes [2, 3]. Alternative TSSs could be results of a gene duplication event followed by the loss of functional exons in the upstream copy and diversification of the two duplicated promoters. Alternative TSS usage can affect gene expression and generate diversity in a variety of ways. On the transcriptional level, alternative TSS could result in tissue-specific expression, temporally regulated expression, and the amplitude of expression. On the post-transcriptional level, alternative TSS can affect the stability and translational efficiency of the mRNA. Furthermore, alternative TSS can result in protein isoforms with a different amino terminus, which can lead to alterations in protein levels, functions, or subcellular distribution. Therefore, the investigation of temporal switching of TSSs can provide insights into the regulation of different protein isoforms, and presumably their differences in function. One way to optimally identify differential use of isoforms is to examine transcriptional regulation over developmental time.

One high-throughput technique to survey gene expression at the transcriptome level is Cap Analysis Gene Expression (CAGE) which generates a genome-wide expression profile based on sequences from the 5′ end of the mRNA [4, 5]. In the FANTOM project, CAGE has been shown to identify different TSSs and the corresponding promoters for single genes [6–9]. With CAGE data, one can infer the TSS usage through the number of transcripts produced at that particular TSS. When more than one TSS is used at a single time point from a single gene, the TSS with highest expression is considered the “dominant” TSS. The understanding of how the TSS usage changes during development can shed light on how a single gene can function differently over developmental stages through temporally regulated alternative mRNA and protein isoforms.

The complexity of brain development requires intricately controlled expression of specific genes across time. The cerebellum is often used as a model in analyses of brain development due to its limited number of major cell types. These cells are positioned in spatially defined territories of the developing cerebellum. The cerebellum has also been the focus of two extensive genome-wide gene expression profiling of the developing cerebellum [10, 11]. Detailed information on temporally regulated promoter usage of developmentally important genes - which is still largely lacking - can provide valuable information on genome diversity. Moreover, different isoforms of these genes may be translated into distinct protein products that perform different tasks. Such analyses would give insight to the alterations made to the form of the final transcript, localization for transcription factors motif prediction, utilization, and associated regulatory network changes. Thus, in collaboration with the FANTOM5 project [12], we generated a CAGE dataset for the developing cerebellum with 12 time points to study temporally-regulated gene expression and alternative TSS usage during cerebellar development.

TSS switching events across samples were systematically identified by comparing differential promoter transcription levels between pairs of TSSs and pairs of developmental time points, and by applying the Silvapulle F_Q_ test, a statistical method for constrained hypothesis testing that we specifically apply for the detection of crossover TSS switching events [13]. The F_Q_ test produces *p*-values to estimate significance of a crossover switching event. We have applied the F_Q_ test to our cerebellar time series to identify novel TSS switching events during cerebellar development. Our hypothesis was that differential TSS usage can result in significant regulatory changes that underlie cellular events critical for cerebellar development and morphogenesis. By taking advantage of the FANTOM5 collaboration with our cerebellar developmental time course, we identified 48,489 novel TSS switching events, including 9767 events in which the dominant TSS shifts over time. These TSS switching events were predicted to produce temporally-specific gene transcripts and protein products that can play important regulatory and functional roles during cerebellar development.

## Methods

### Mouse colony maintenance and breeding

This research was performed with ethics approval from the Canadian Council on Animal Care and research conducted in accordance with protocol A12–0190. C57BL/6 J mice were used in all experiments and were imported from The Jackson Laboratory (Maine, US) and maintained in our colony as an inbred line. To standardize the time of conception, timed pregnancies were set up. Every weekday at 10:00 am, females were coupled with male; at 3:00 pm, the females were checked for vaginal plugs and removed from their partners. The appearance of a vaginal plug was recorded as the day of conception (i.e. embryonic day 0) and embryos were collected at 10 am on embryonic day 11–18 (E11-E18) every day and postnatal day 0–9 (P0-P9) every 3 days for a total of 12 time points in our cerebellar time series.

### Tissue processing

On the day of embryo collection, the mothers were sacrificed and embryos were removed from the uterus in ice-cold RNAse-free PBS. Cerebella were dissected from the head of the embryos, then pooled with littermates, and snap-frozen in liquid nitrogen. Three replicate pools of whole cerebella samples were collected at each time point. The standard TRIzol RNA extraction protocol [14] was used for tissue homogenization and RNA extraction.

### Quality assessment

A Bioanalyzer (Agilent, Santa Clara, CA) was used to examine RNA quality. All RNA samples used for the time series achieved high RNA Integrity (RIN) scores above 9.0. The samples were sent to RIKEN Omics Center at Yokohama, Japan, as part of Functional Annotation of the Mammalian Genome 5 (FANTOM5) collaboration for CAGE analysis.

### Transcriptome library generation by HeliScopeCAGE

**Fig. 1:**
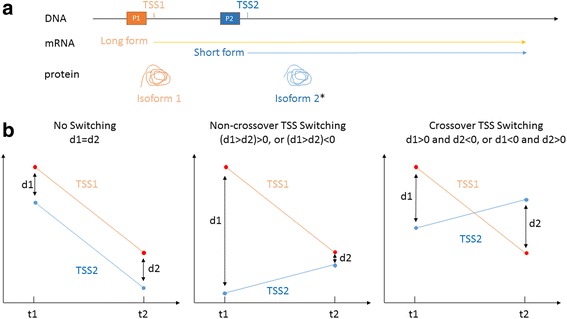
A schematic diagram of alternative transcription start sites (TSSs) and the classes of TSS switching. **a** Alternative TSSs can generate different splicing variants that can be translated into different protein isoforms. *the functional domains may be affected by alternative TSSs which results in functional diversity. **b** Different outcomes comparing alternative TSS usage at two time points – no TSS switching, non-crossover TSS switching or crossover TSS switching. Y-axis represents the quantitative measure of TSS usage measured by the expression level of its mRNA transcript. X-axis represents the two developmental time points used in the comparison (t1 vs. t2)

CAGE is a technique that generates a genome-wide expression profile based on sequences from the 5′ end of the mRNA. With CAGE, the first 27 bp from the 5′ end of RNAs were extracted and reverse-transcribed to DNA [4]. The short DNA fragments were then systematically sequenced with the Helicos platform [15]. Each sequenced tag was then mapped to the reference genome to identify the transcription start site (TSS) of the gene from which it was transcribed. “Tag per million” (tpm) was used as a measure of the expression level of RNAs based on concentration – an expression of “10tpm” means that out of each million total transcripts, 10 were transcribed from the TSS in question. Alternative TSSs (illustrated in Fig. 1a) can be detected when multiple CAGE tags are mapped to the same gene locus in the reference genome. Mapped CAGE tags can be clustered into promoter regions after thresholding to determine bona fide promoter regions in the genome. For this analysis, we use the list of promoter regions published by the FANTOM5 Consortium [5].

### TSS switch detection

TSS switching events are detected by comparing the expression of transcripts from two TSSs of a single gene at two time points. The difference in expression level of the two TSSs is designated d1 and d2 at time point 1 and time point 2, respectively. The null hypothesis is that there is no switching for the two TSSs (d1 = d2, see Fig. 1b). The test of this hypothesis was performed using the standard t-test, with candidate switching events identified at this preliminary stage if the adjusted *p*-value was <0.2. A non-crossover TSS event is detected if one TSS is used more frequently at one time point compared to the other, but the same TSS is used dominantly at both time points (d1 > d2, or d1 < d2, both d1 and d2 same sign, Fig. 1b). A crossover TSS switching event is detected if one TSS is used more frequently at one time point compared to the other, and that the dominant TSS switches at the two time points (d1 > 0 and d2 < 0 or d1 < 0 and d2 > 0, Fig. 1b). In order to reduce potential confounding of TSS switching events by differential aggregate promoter expression between time points, candidate events were further limited to TSS pairs that do not change in overall mean expression between developmental stages being compared. The null hypothesis tested at this stage is that the mean TSS expression at the two time points is equal, and results were filtered out if the t-test adjusted *p*-value was <0.1.

In addition to the differences in expression (d1,d2), the results of TSS switching are represented using the F_Q_ statistic [12] which formally tests for the presence of crossover switching for each gene. The test of the null hypothesis of no differential crossover promoter usage corresponds to a test involving the F_Q_ statistic, which is functionally similar to the ANOVA F-test. Exact *p*-values for this test are obtained as described in Silvapulle [12]. To our knowledge, the Silvapulle F_Q_ test is the only statistical test available that was specifically developed for testing hypotheses regarding qualitative interaction, and which we apply in the current study for testing the presence of crossover switching in gene promoter usage.

All *P*-values are adjusted for multiple comparisons using the Benjamini–Hochberg method to control the false discovery rate. The *P*-value of the F_Q_ test was used as an indicator of significance for choosing biological validation candidates.

### Gene ontology analysis for gene with crossover switching events

To identify cellular processes and molecular pathways in genes with crossover TSS switching events, we used Database for Annotation, Visualization and Integrated Discovery program (DAVID, https://david.ncifcrf.gov/ [16]) to examine the gene ontology of genes with at least one crossover event with *p* < 0.05 in F_Q_ test. Top 20 GO terms were used for overall analysis in crossover TSS switching genes during cerebellar development. Furthermore, for temporal functional analysis of crossover TSS switching events, top 20 GO terms were generated with DAVID for all events associated with three developmental time points – E13, E15 and P0.

### In silico validation of gene expression with established databases and experimental validation with gene structure prediction and quantitative real-time PCR

We used online databases to examine the 20 genes with the lowest *p*-values. First, we used in situ resources - Genepaint (http://genepaint.org [17]) and Allen Brain Atlas (http://www.brain-map.org [18]) to examine the genes’ expression in the cerebellum. Second, we examined the predicted mRNA structures from the two TSSs with the intron/exon database Aceview (http://www.ncbi.nlm.nih.gov/IEB/Research/Acembly/ [19]) as well as functional domains of their protein products from protein domain database PhosphoSitePlus (http://www.phosphosite.org [20]) to determine the potential effect of TSS switching events on biological function.

Three genes were chosen for further validation for TSS-specific quantitative real-time PCR for the validation of alteration in TSS usage at E12, E15 and P9. Cerebellar RNA was extracted from C57BL/6 J mice at E12, E15 and P9 following the same procedure that were used for HeliScopeCAGE RNA collection. cDNAs were produced with random hexamers using the High Capacity cDNA Archive Kit (Applied Biosystems). cDNA products were diluted to 100 ng total RNA input. Sequences of the transcript of interest were loaded into Primer Express® software (Applied Biosystems). For each gene, an isoform-specific forward primer was designed for each of the long and short isoform, while the reverse primer aligns to a common sequence that is shared by both isoforms. Amplicon lengths were between 80 and 120 bp. The qPCR was performed with the FAST SYBR Green PCR Master Mix (Applied Biosystems) on an ABI StepOne Plus Sequence Detection System (Applied Biosystems). All runs were normalized to the control gene, Gapdh. Three biological replicates were prepared for each gene target and three technical replicates were performed for each biological replicate. Gene expression was represented as relative quantity against the negative control which used water as the template (noted as “Relative Quantity vs. H2O” in figures). The results of Real-Time PCR were analyzed and graphed by ABI StepOne Plus Sequence Detection System (Applied Biosystems). The expression data were compared with the HeliScope-CAGE data.

## Results

### Overview of promoter switch events during cerebellar development

**Fig. 2:**
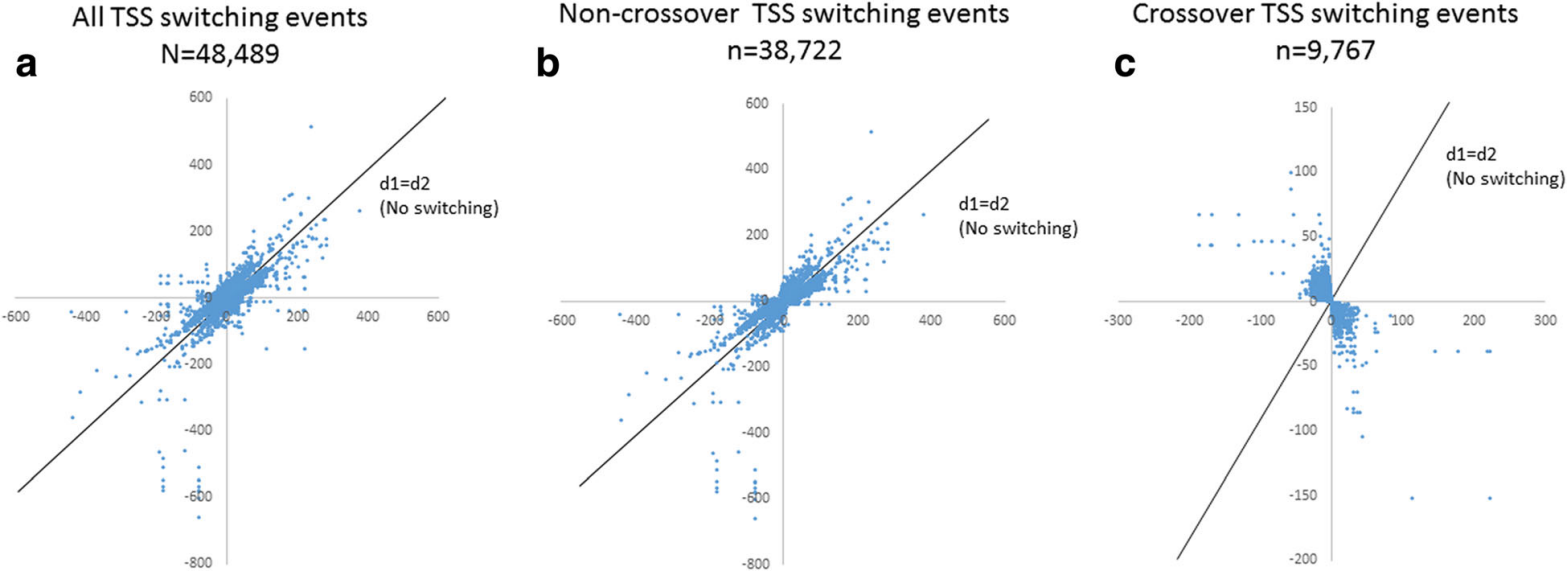
Overview of TSS switching events during cerebellar development. **a** Overview of 48,489 TSS switching events during cerebellar development. These events significantly deviate from the no-switching line (indicated by d1 = d2) (*p* < 0.05). **b** Overview of 38,722 non-crossover TSS switching events during cerebellar development. **c** Overview of 9767 crossover TSS switching events during cerebellar development. X-axis represents d1, which is the difference in expression between the two TSSs, measured in tags per million (tpm), at developmental time point 1 (t1), see Fig. 1b for a graphic illustration. Y-axis represents d2, which is the difference in expression between the two TSSs, measured in tag per million (tpm) at developmental time point 2 (t2), see Fig. 1b for a graphic illustration

Our cerebellar time series, which consisted of transcriptome data from 12 time points, yielded a total of 183,903,557 CAGE tags that are mapped to 25,207 genes in the reference genome. We identified 48,489 TSS switching events (Fig. 2a) in the cerebellar time series data that occur in 5433 genes. These events are comprised of 38,722 non-crossover switching events (Fig. 2b) that occur in 5293 genes, and 9767 crossover switching events (Fig. 2c) that occur in 1511 genes. One thousand three hundred seventy-one out of 1511 genes (~91%) that have crossover TSS switching events also have at least one non-crossover switching event. This indicates that crossover TSS switching events are rarer and occur in fewer genes when compared to the non-crossover events.

When comparing the cerebellar TSS switching data to nine other tissues in the FANTOM5 dataset (see Table 1; detailed descriptions about time series on these tissues can be found in [12]), our cerebellar development time series has the 3rd highest total number of TSS switching events (48,489) behind “Epithelial to mesenchymal” (132,661 events) and “Adipocyte differentiation” (66,087 events); and is the highest of the three samples derived from ectoderm [“Human iPS to neuron (wt) 1” and “Trachea epithelia differentiation”]. While the cerebellar development time series has less total events than “Epithelial to mesenchymal” and “Adipocyte differentiation” samples, it has a higher frequency of crossover TSS switching events - 20.1% vs 17.6 and 12.5%, respectively. Interestingly, when compared to 48,489 events found in the cerebellum, four out of the five remaining datasets had a higher percentage of crossover events but a much lower number of total switching events.

**Table 1:** Comparison of TSS switching events during cerebellar development with other FANTOM5 datasets

Time Series	Germ layer	TP#	Switching#	Gene#	%	Non-Xover	%	Xover	%
Cerebellar development	Ectoderm	12	48,489	5433	21.6	38,722	79.9	9767	20.1
Human iPS to neuron (wt) 1	Ectoderm	4	45,069	6692	26.5	41,302	91.6	3767	8.4
Trachea epithelia differentiation	Endoderm	19	8389	2458	9.8	6112	72.9	2277	27.1
Adipocyte differentiation	Mesoderm	16	66,087	5996	23.8	57,857	87.5	8230	12.5
Epithelial to mesenchymal	Mesoderm	21	132,661	7004	27.8	109,252	82.4	23,409	17.6
BMM TB activation IL13	Mesoderm	11	825	527	2.1	564	68.4	261	31.6
AoSMC response to IL1b	Mesoderm	10	192	159	0.6	129	67.2	63	32.8
Macrophage response to LPS	Mesoderm	23	32,234	4557	18.1	22,239	69.0	9995	31.0
ES to cardiomyocyte	Mesoderm	13	189	163	0.6	100	52.9	89	47.1
Myoblast to myotube	Mesoderm	9	21,912	4249	16.9	18,735	85.5	3177	14.5

*TP#* number of time points in the time series, *Switching #* total number of TSS switching events found in the dataset, *Gene #* total number of genes with at least one TSS switching event, *Column 6: %* TSS switching genes over all 25,207 genes, *Non-Xover* total number of non-crossover TSS switching events found in the dataset, *Column 8: %* percentage of non-crossover events over all switching events, *Xover* total number of crossover TSS switching events found in the dataset, *Column 10: %* percentage of crossover events over all switching events

In conclusion, cerebellar development showed a high frequency in crossover TSS switching among datasets with a high number of total switching events.

### Distribution of TSS switching events in cerebellar transcriptome

**Fig. 3:**
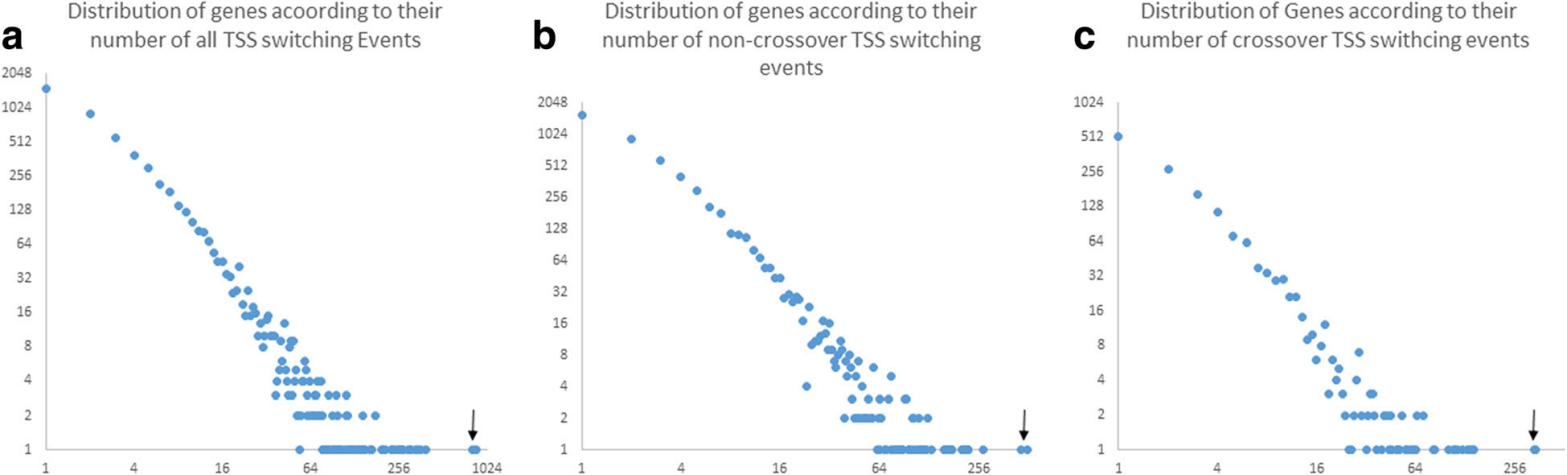
Distribution TSS switching events in different genes during cerebellar development. **a** Distribution 48,489 TSS switching events in genes during cerebellar development. Arrow points to the two genes with more than 800 switching events. **b** Overview of 38,722 non-crossover TSS switching events in 5293 genes during cerebellar development. **c** Overview of 9767 crossover TSS switching events in 1511 genes during cerebellar development. x-axis – number of TSS events occurs within one gene (log2 scaled). y-axis – number of genes that have the number of TSS events indicated on the x-axis

When we looked at the distribution of the 48,489 TSS switching events over the 5433 genes, we found a majority of genes with few events and a minority of genes with many events. Thus, we found there are 1534 (28% of TSS switching gene) genes with one TSS switching event; and only two genes with more than 800 switching events (Fig. 3a). When we looked at the top 20 genes with the most TSS events (listed in Table 2), we observed that these genes account for 13.5% for all TSS switching events, or a total of 6567 events. From Fig. 3 (as well as Table 2), we can see that there are two outlier genes that have the largest number of TSS switching events for all 3 groups (all TSS, non-crossover and crossover, indicated by arrows in Fig. 3a-c) - Frmd4a (FERM domain containing 4A) with a total of 852 TSS switching events and Ank3 (ankyrin 3) with a total of 801 TSS switching events (see Table 2). These two genes have more than twice the number of TSS switching events than the next closest gene, Abr (active BCR-related gene) with a total of 386 TSS switching events. The numbers of TSS switching events are more evenly distributed across the rest of the 18 genes with a higher frequency of switching (see Fig. 3) as the difference between each rank is less than 10% of the number of events in this group.

**Table 2:** Top 20 genes with highest numbers of TSS switching events

	Gene ID	All events	Non-crossover events	Crossover events
1	Frmd4a	852	509	343
2	Ank3	801	464	337
3	Abr	386	275	111
4	Ednrb	356	211	145
5	Iqsec1	348	206	142
6	Bcat1	329	221	108
7	Pde4d	308	176	132
8	Ldb1	304	167	137
9	Sorbs2	297	175	122
10	Cnpy1	273	158	115
11	Dlg2	266	223	43
12	Ebf1	262	160	102
13	Ablim1	254	204	50
14	Zeb2	246	218	28
15	Trim2	233	168	65
16	Celf2	227	162	65
17	Map2	226	170	56
18	Itgb8	208	126	82
19	Ank2	197	126	71
20	Ptprg	194	111	83

When comparing the distribution of crossover and non-crossover events, we found that crossover switching events are clustered in fewer genes when compared with non-crossover events. Since the frequency of non-crossover switching is about four times the number of cross-over (38,722:9767 or 3.96:1), we would expect roughly a 4:1 ratio for non-crossover: crossover events for any given gene, assuming an even distribution of both categories. Indeed, we observed roughly a 4:1 ratio for Ablim1 (204 non-crossover events and 50 crossover events) and Dlg2 (223 non-crossover events and 43 crossover events, Table 2). However, for the majority of the 20 genes with the greatest number of switching events, the frequency of crossover events is much higher than one fourth of the non-crossover counterpart, such as the two outlier genes mentioned above - Frmd4a (509 non-crossover events vs 343 crossover events) and Ank3 (464 non-crossover events vs 337 crossover events, Table 2). This un-even distribution of crossover events is also reflected by the lower abundance of genes with a low number of switching events – 3052 genes have less than 3 non-crossover events (Fig. 3b) and only 944 genes have less than 3 crossover events (Fig. 3c). In conclusion, we found that crossover events tend to cluster in a fewer number of genes when compared to the non-crossover counterpart.

### Gradual increment in the number of crossover TSS switching events over developmental time

Next, we focused in the temporal distribution of crossover TSS switching. When we look at a day-to-day change in promoter usage (E12 vs E11, E13 vs E12 etc., underlined in Table 3), TSS switching occurs evenly across cerebellar development from 13 events to 39 events - with the exception of the E13-E12 comparison (Table 3). There are 93 TSS switching events between E12 and E13 indicating a major shift in promoter usage at this developmental stage.

**Table 3:** Distribution of crossover TSS switching events across time in cerebellar development (*N* = 9767)

	E12	E13	E14	E15	E16	E17	E18	N0	N3	N6	N9
E11	**31**	180	290	340	320	333	236	291	354	279	429
E12		**93**	159	200	209	238	162	228	274	225	381
E13			**21**	59	99	97	76	118	190	180	327
E14				**34**	55	86	69	114	203	198	303
E15					**35**	30	29	56	129	143	301
E16						**29**	23	53	103	113	226
E17							**13**	39	58	91	204
E18								**20**	42	76	123
N0									**39**	60	134
N3										**25**	76
N6											**17**

Number of crossover TSS switching events that are found in adjacent time points are in Bold

For example, **93** in column 3, row 3 represents 93 crossover events found between E12 and E13

To examine the general pattern of TSS switching during cerebellar development, we counted promoter switch events by developmental time points (Table 3). Among the 12 data points in our time course, a total of 66 comparisons between two data points have been carried out to search for the switching of alternative TSSs (Table 3). Over the time series, there is a general incremental number of crossover switching events that are detected between two samples that are temporally distant. This most likely reflects the gradual shift of cerebellar transcriptome and TSS usage during development. There are rare exceptions to this pattern, for example, there are more switching events between E11 and E17 samples than found between E11 and E18 samples.

### Gene ontology analysis for genes with the most significant crossover TSS switching events

**Fig. 4:**
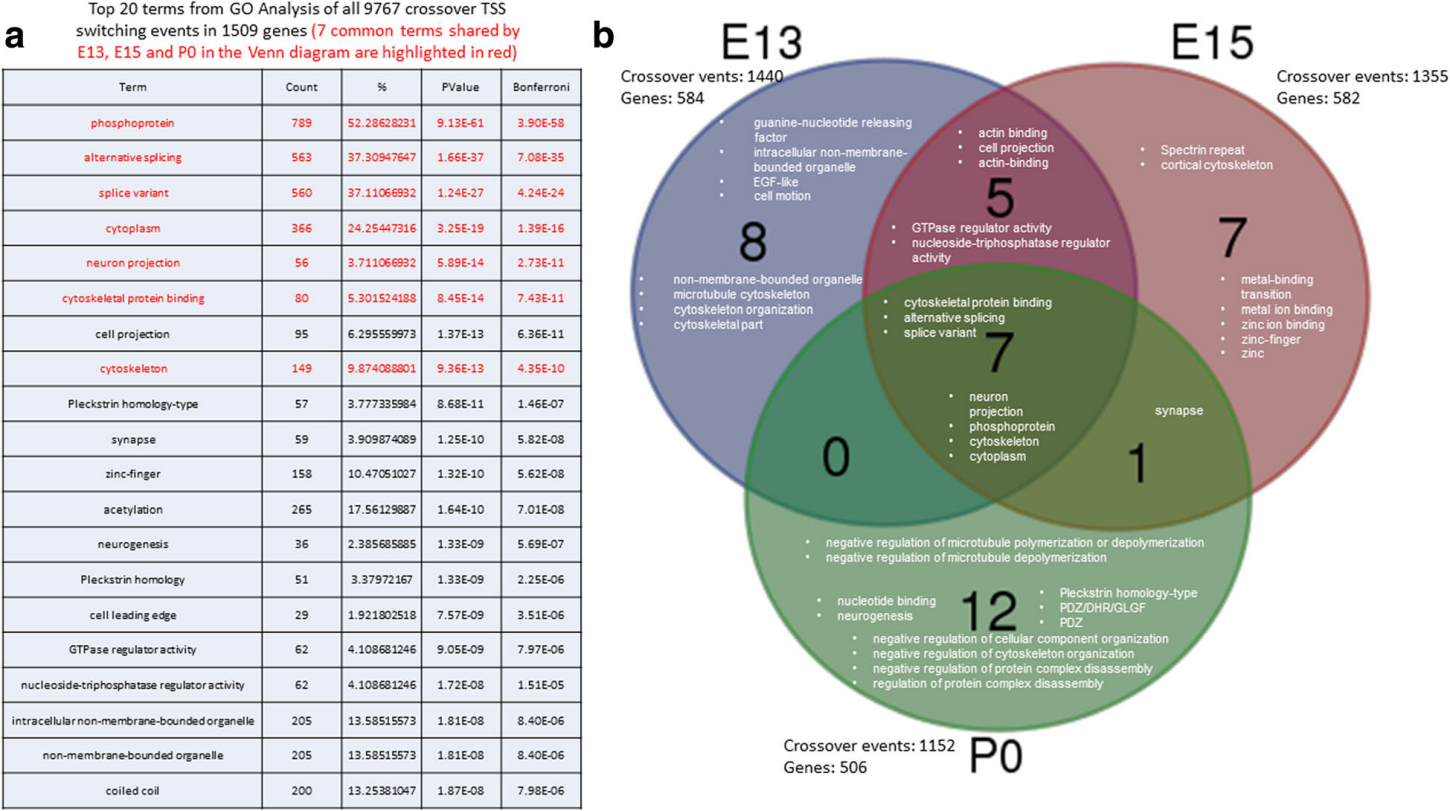
GO Analysis for genes significant (*p* < 0.05) for crossover switching at all time points (*left*) and at three selected time points (*right*). **a** Top 20 terms from GO analysis of all 9767 crossover TSS switching events in 1509 genes For column heading: “Term” is the GO term, “Count” is the number of genes associated with the GO term and “%” is the fraction of the number of genes associated with the GO term divided by the total input of 1509 genes, “PValue” and “Bonferroni” represent the significance of the GO term. **b** A Venn diagram comparing the top 20 GO terms from crossover TSS switching events between all samples and either E13, E15 or P0 samples

To functionally annotate the genes that undergo significant crossover TSS switching, we used the Database for Annotation, Visualization and Integrated Discovery program (DAVID, https://david.ncifcrf.gov/ [16]) to examine the biological process and terms associated with crossover TSS switching genes. From 1509 genes with 9767 crossover TSS switching events at *p* < 0.05, we analyzed 20 gene ontology (GO) terms with the lowest *p*-value from the DAVID analysis (see Fig. 4a). Terms associated with neuronal development, such as “neuron development”, “neuron projection” and “synapse” also showed up at high significance levels from DAVID analyses (Fig. 4a).

We have found that the largest alteration in gene expression occurs at E13, E15 and P0 (manuscript in preparation) and were interested to determine the extent that crossover TSS switching plays a role in transcriptome diversity. When comparing crossover events at E13 with all other time points we find 1440 significant (*p* < .05) events in 584 genes. When comparing crossover events at E15 with other time points we find 1355 significant (*p* < 0.05) events in 582 genes. Finally, when comparing crossover events at P0 with all other time points we find 1152 significant (*p* < .05) events in 506 genes. We used these gene lists as input to DAVID and the top 20 terms were selected for these temporal comparisons among the three time points (Fig. 4b). We found that 7 terms (phosphoprotein, alternative splicing, splice variant, cytoplasm, neuron projection, cytoskeletal protein binding and cytoskeleton) were shared among each of the three time points. These 7 GO terms were also found among the 8 most significant terms in the analysis with all genes discussed previously. We also observed that comparisons between shorter time spans yield more common GO terms –e.g., there are 5 terms shared between genes with crossover TSS events at E13 and E15, 1 term between E15 and P0 and no terms were common between E13 and P0. Lastly, the majority of GO terms unique to a given time point shared a common theme that may reflect active biological process occurring at the given time – e.g., four out of eight E13 terms were associated with cell motion and cytoskeleton; five out of seven E15 terms were associated with ion binding and six out of twelve P0 terms were associated with regulation of intracellular organization.

### Validation of promoter switching events

To further investigate the genes with the 20 most significant TSS switching events, we used the in situ hybridization expression database Genepaint (http://www.genepaint.org/) to examine their expression pattern in the cerebellum (summarized in Table 4). Three of these genes showed robust cerebellar expression (Gpc6, Anp32a and Cntnap2) and were chosen to demonstrate the potential biological roles of the TSS switching events during cerebellar development. First, their mRNA structures were obtained from the intron/exon database Aceview (http://www.ncbi.nlm.nih.gov/IEB/Research/Acembly/); then their protein structure for each isoform was obtained from protein domain database PhosphoSitePlus (http://www.phosphosite.org); finally, the TSS switching events for these three genes were validated with quantitative real-time PCR with promoter-specific primers.

**Table 4:** Cerebellar expression patterns of genes with most significant switching events at E14.5 from the in situ database, Genepaint

Gene	Full name	Genepaint
DLG3	discs, large homolog 3	N/E
SLC12A5	solute carrier family 12, member 5	N/E
PDE4D	phosphodiesterase 4D	NE, interior cerebellum
IQSEC1	IQ motif and Sec7 domain 1	N/E
CNTNAP2	contactin associated protein-like 2	RL specific
CNPY1	canopy 1 homolog	N/A
MAPK8IP1	mitogen activated protein kinase 8 interacting protein 1	specific cerebellar nuclei, spinal cord
DLGAP4	discs, large homolog-associated protein 4	widespread cerebellum
ANK3	ankyrin 3, epithelial	interior cerebellum
CACNB4	calcium channel, voltage-dependent, beta 4 subunit	N/E
ANP32a	acidic (leucine-rich) nuclear phosphoprotein 32 family	strong, EGL & NE specific staining
TMX3	thioredoxin-related transmembrane protein 3	N/A
APBB3	amyloid beta (A4) precursor protein-binding, family B, member 3	N/E
PRMT8	protein arginine N-methyltransferase 8	widespread cerebellum
EDNRB	*Mus musculus* endothelin receptor type B	strong NE specific staining
SEMA4G	sema domain 4G	widespread cerebellum
FBLN5	fibulin 5	N/E
ZRANB1	zinc finger, RAN-binding domain containing 1	N/E
ZBTB38	zinc finger and BTB domain containing 38	N/A
IBTK	inhibitor of Bruton agammaglobulinemia tyrosine kinase	N/E
GPC6	glypican 6	Strong NE, NTZ specific staining
HSPH1	heat shock 105 kDa/110 kDa protein 1	N/A
ZFP451	Mus musculus zinc finger protein 451	moderate EGL staining
GRAMD1B	GRAM domain containing 1B	N/E

*N/E* not expressed or ineffective probe, *NE* neuroepithelium, *RL* Rhombic lip, *EGL* external granular layer, *NTZ* nuclear transitory zone, *N/A* data not available

**Fig. 5:**
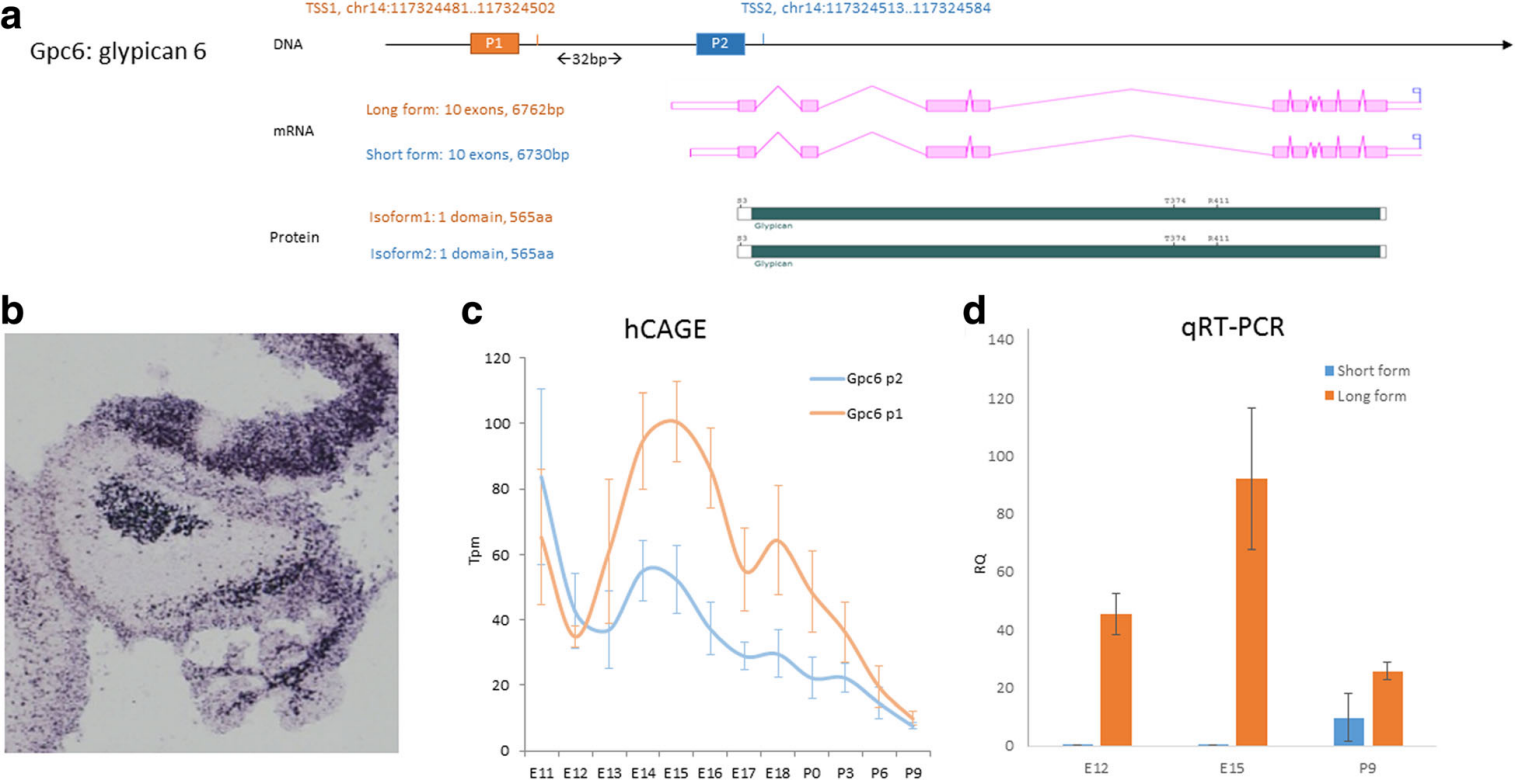
Alternative TSSs in glypican 6 (Gpc6) and experimental validation of its non-crossover switching events with Real-time PCR. **a** Schematic DNA structure of Gpc6, alternative mRNA variants and un-altered protein structure. **b** in situ expression of Gpc6 in mouse cerebellum at E14.5 (from GenePaint). **c** HeliscopeCAGE expression data for the two alternative TSSs during cerebellar development. X-axis: time, from embryonic day 11 (E11) to postnatal day 9 (P9). Y-axis: expression level measured in tpm (tags per million). **d** qRT-PCR expression data demonstrating a non-crossover TSS switching event between E15 and P9. X-axis: time at E12, E15 and P9. Y-axis: expression level measured in RQ (relative quantity against H2O as negative control)

When we investigate the role of the most significant TSS switching events, we found that some of the most significant events do not seem to affect protein sequence and may play roles in transcriptional or post-transcriptional regulation. One example we examined is Glypican-6(Gpc6) - a member of Glypican family that is found on the cell surface and plays important roles in cellular growth control and differentiation. The two TSS sites are 32 bp apart in the genome and mRNA that originate from the two TSS sites differ in the first exon in the 5’UTR region (Fig. 5a). The two forms of mRNA were predicted to be translated into the same protein isoform that contains 565 amino acids. The single glypican domain that makes up the majority of the peptide is not effected by the TSS switching event (Fig. 5a). Therefore, the usage of alternative TSSs in Gpc6, which is expressed in the NE, NTZ and EGL in the cerebellum (Fig. 5b), could play a regulatory role, such as temporally regulated expression, amplitude of expression, mRNA stability and mRNA translational efficiency. Our qRT-PCR data confirmed the TSS switching prediction (Fig. 5c) and showed that it undergoes a non-crossover TSS switching between E15 (TSS2 is the dominant form and has >2 fold usage compared with TSS1) and P9 (TSS2 has slightly higher usage than TSS1, but remains as the dominant form, see Fig. 5d).

**Fig. 6:**
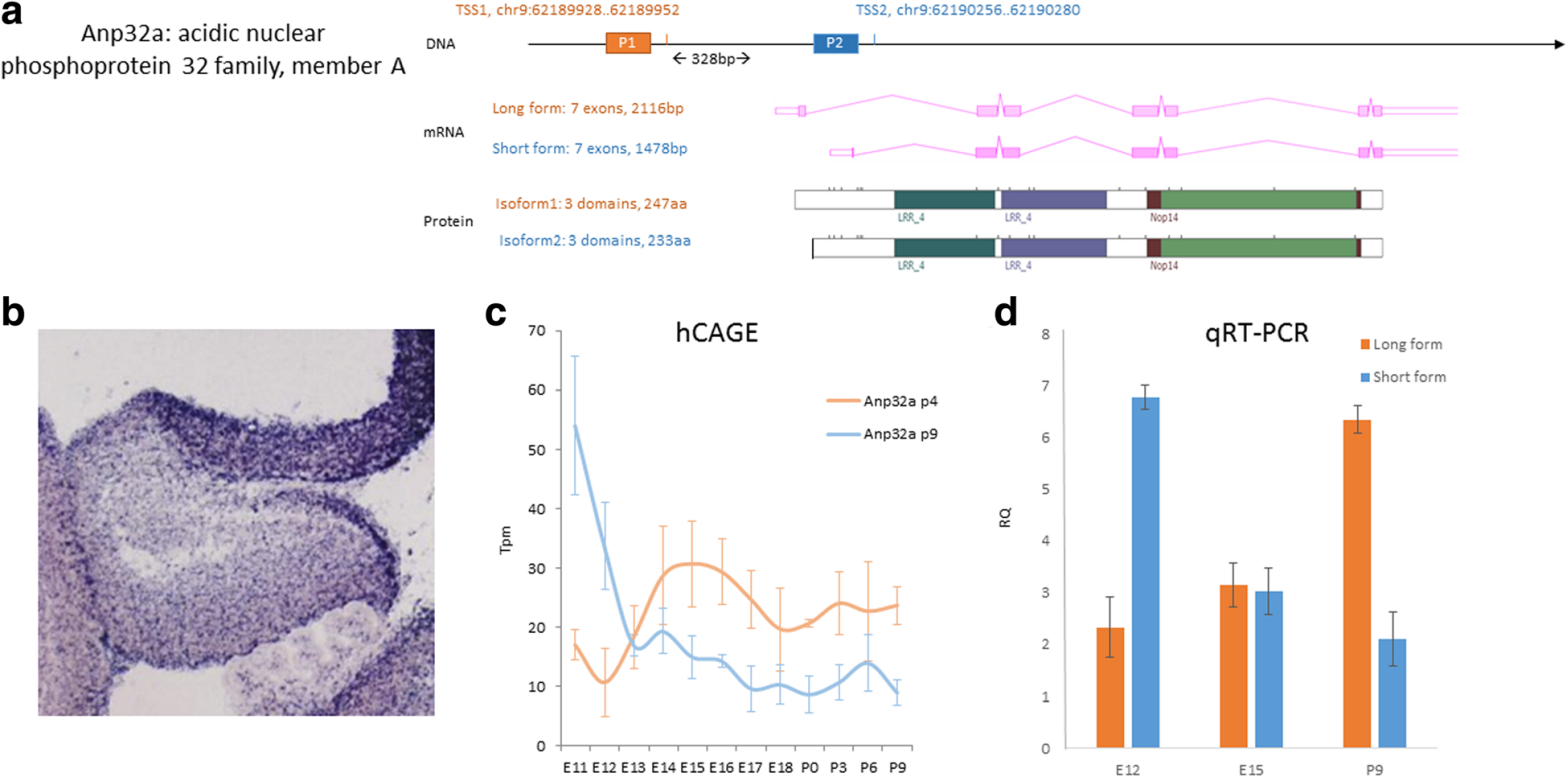
Alternative TSSs in Acidic nuclear phosphoprotein 32 family, member A (Anp32a) and experimental validation of its crossover switching events with Real-time PCR. **a** Schematic DNA structure of Anp32a, alternative mRNA variants and altered protein structure at the N-terminus. **b** in situ expression of Anp32a in mouse cerebellum at E14.5 (from GenePaint). **c** HeliscopeCAGE expression data for the two alternative TSSs during cerebellar development. X-axis: time, from embryonic day 11 (E11) to postnatal day 9 (P9). Y-axis: expression level measured in tpm (tags per million). **d** qRT-PCR expression data demonstrating a crossover TSS switching events between E12 and P9. X-axis: time at E12, E15 and P9 Y-axis: expression level measured in RQ (relative quantity against H2O as negative control)

Some of the most significant TSS switching events occur between two TSSs that could produce protein isoforms with different N-termini, which may or may not affect the function of the protein isoforms. An example of this would be Acidic (leucine-rich) nuclear phosphoprotein 32 family member A (Anp32a) - a member of acidic nuclear phosphoprotein 32 kDa (Anp32) family. The two TSS sites are 328 bp apart in the genome and mRNA that originate from the two TSS sites differs in the first exon in the 5′UTR region as well as the N-terminus of protein products. The first 12 amino acids of the long isoform were absent on the short isoform. Functional domains were not affected by the TSS switching event - both isoforms retained two LRR4 domains and a single NOP14 domain (Fig. 6a). The difference at the N-terminus can lead to alterations in Anp32a’s protein level, subcellular distribution or function in the EGL where it is strongly expressed (Fig. 6b). As predicted (Fig. 6c) and validated with our qRT-PCR data, Anp32a undergoes a crossover TSS switching between E12 (TSS9 as dominant form) and P9 (TSS 4 as dominant form, see Fig. 6d).

**Fig. 7:**
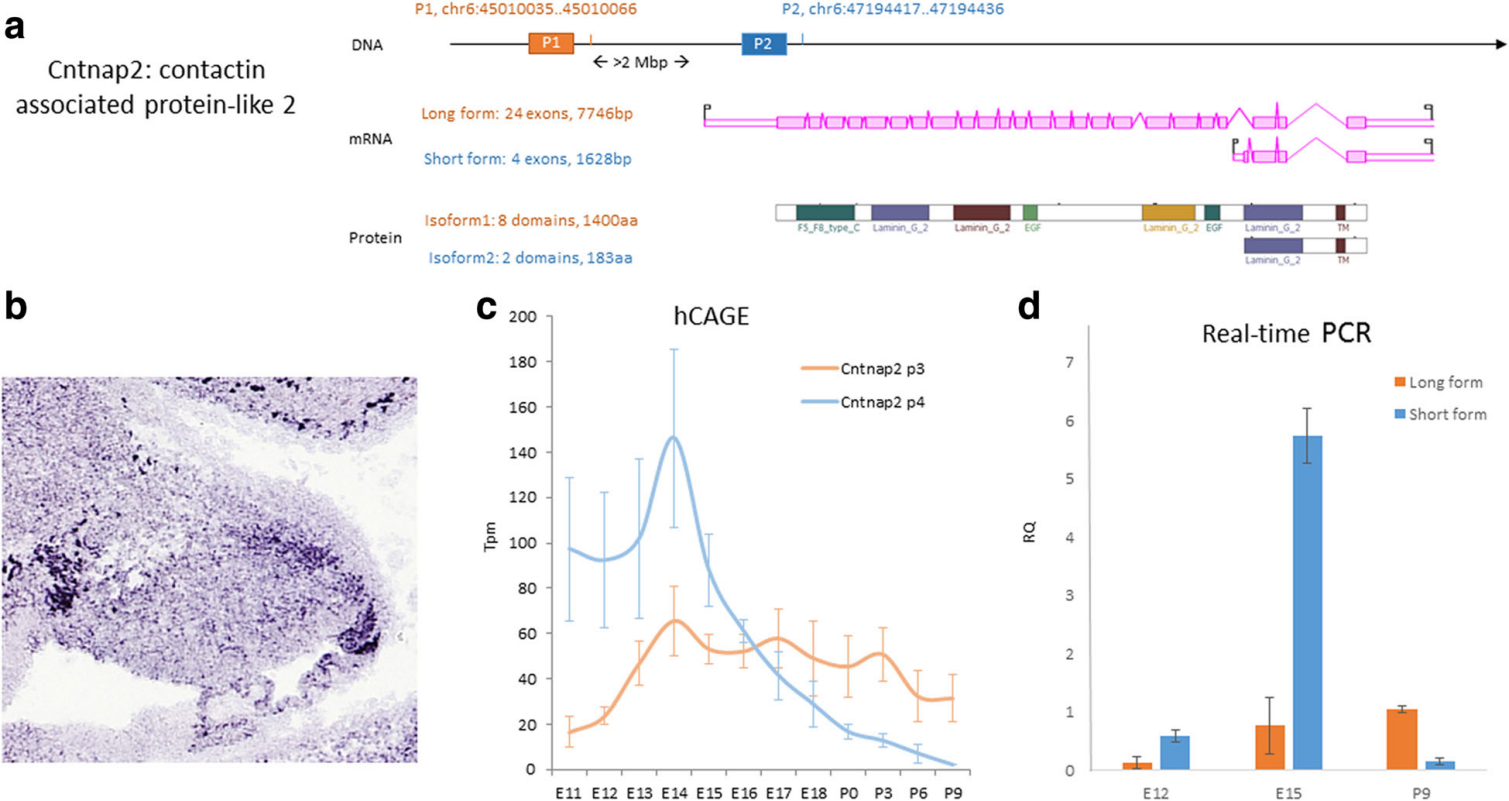
Alternative TSSs in contactin associated protein-like 2 (Cntnap2) and experimental validation of its crossover switching events with Real-time PCR. **a** Schematic DNA structure of Cntnap2, alternative mRNA variants and truncated protein structure of the short isoform. **b** in situ expression of Cntnap2 in mouse cerebellum at E14.5 (from GenePaint). **c** HeliscopeCAGE expression data for the two alternative TSSs during cerebellar development. X-axis: time, from embryonic day 11 (E11) to postnatal day 9 (P9). Y-axis: expression level measured in tpm (tags per million). **d** qRT-PCR expression data demonstrating a crossover TSS switching events between E12 (as well as E15) and P9. X-axis: time at E12, E15 and P9. Y-axis: expression level measured in RQ (relative quantity against H2O as negative control)

Lastly, among the genes with the most significant TSS switching events, we have discovered a crossover TSS switching event where protein function is highly affected in the Contactin-associated protein-like 2 (Cntnap2) – a gene encodes a member of the neurexin family which functions as cell adhesion molecules and receptors in neurons. The two TSS sites, that lead to the transcription of two NCBI-validated mRNA refseqs, are more than 2 million bp apart in the genome. mRNAs that originate from the two TSS sites differ by more than 6000 bp and consist of the first 20 exons of the long mRNA – only 4 exons at the 3′ end of the long form mRNA (NCBI Locus: NM_001004357.2) are present in the short form (NCBI Locus: NM_025771.3, see Fig. 7a). The Cntnap2 protein, in its long isoform (NCBI Locus: NM_025771.3), contains 1400 amino acids and many functional domains including one F5/8 type C domain, two epidermal growth factor repeats domains, four laminin G domains and a TM domain. The short protein isoform of Cntnap2 (NCBI Locus: NP_080047.1), which has 190 amino acids has only two of the eight functional domains remaining, the last laminin G domain and the TM domain (Fig. 7a). In the Genepaint database, a probe specific to the long isoform of Cntnap2 was used, and it is indicated that the long isoform is primarily expressed in the rhombic lip of the cerebellum at E14.5 (Fig. 7b). According to our prediction (Fig. 7c) and qRT-PCR results, Cntnap2 undergoes a crossover TSS switching between E15 (TSS4 as dominant form) and P9 (TSS3 as dominant form, see Fig. 7d). The highly differentiated protein isoforms of Cntnap2 suggest the gene’s temporal shift in protein functions during cerebellar development where a truncated form is made specifically in the during early embryonic stages.

## Discussion

### High prevalence of alternative TSSs in mammalian genomes

In this study, we have identified 5293 genes (~21% of a total of 25,207 genes) that exhibit differential TSS usage during cerebellar development. These findings are in line with previous studies and indicate that TSS switching events are common and can play an important role in the diversity of the cerebellar transcriptome during development [21–23]. Furthermore, we have identified 9767 crossover TSS switching events which suggests an alteration in the dominant TSS over time. Since the alternative mRNA isoforms could be translated into functionally different products, a crossover switching event suggests that one gene can play different roles at different time points in development.

Alternative usage of multiple TSSs of one gene is common in mammalian genomes. It is a key mechanism to increase mRNA and protein diversity since multiple mRNAs from a single gene can encode distinct protein isoforms with different functions (reviewed in [24]). Recent studies suggest that about half of the mouse genes have multiple alternative promoters [25, 26]. For example, alternative promoters have been identified in >20% of genes in ENCODE (http://genome.ucsc.edu/ENCODE/) regions [6]. Other genomic studies also found more than a quarter of human genes having multiple active promoters [27–29]. The complex transcriptional regulation of alternative promoter usage has been identified in several genes [24]. Furthermore, in some genes, such as tumor protein p53 (TP53) and guanine nucleotide binding protein (GNAS), alternative promoters were shown to be activated or silenced [29]. However, the focus of previous studies has been the tissue-specific transcriptional regulation of alternative promoters; the temporal aspect of alternative promoter usage during cerebellar development has been overlooked. Our analyses focused on the switching usage of alternative promoter in the mouse cerebellum, and this is the first systematic study of alternative promoter usage in the development of the mouse cerebellum.

### Temporal regulation of alternative TSS associated with developmental processes in the cerebellum

Alternative TSSs reflect different promoter regions that can be used for tissue-specific and/or temporal-specific expression. For example, albumin in hepatocytes has several cis-acting elements that recruit different sets of trans-acting factors, which enable spatial, temporal and dynamics regulation of the transcription of albumin mRNA [30]. In this study, we have identified 9767 crossover TSS switching events in 1511 genes. Thus, in ~20% of genes there is more than one promoter that is used dominantly during cerebellar development. Functional annotation analysis for these genes revealed GO terms that are expected to be associated with alternative promoter usage, such as “alternative splicing” and “splicing variants”, as well GO terms that point to processes where promoter switching might play a role during development, such as “phosphoprotein”, “cytoskeleton organization” and “neuron projection”. Phosphoproteins are involved in the post-translational regulatory process phosphorylation, in which a phosphate group is added to a peptide. The physical binding of phosphoproteins, such as Fas-activated serine/threonine phosphoprotein (FAST), to regulators of alternative splicing has been evidenced by yeast two-hybrid screening and biochemical analyses [31]. Furthermore, the sensory, motor, integrative, and adaptive functions of neuron projections are associated with the development of a growth cone, which is composed primarily of an actin-based cytoskeleton [32]. One of the cytoskeleton remodeling genes, Disabled-1 (Dab1), has multiple isoforms, as a result of alternative splicing [33], that are activated by tyrosine-phosphorylation and play important roles in neuronal positioning by recruiting a wide range of SH2 domain-containing proteins and activates downstream protein cascades through the Reelin signalling pathway [34]. Deficiency in Dab1 pathway resulted in a delay in the development of Purkinje cell dendrites and dysregulation of the synaptic markers of parallel fiber and climbing fiber in the cerebellum [35].

The dominant TSS usually switches gradually over time so that only 3.7% of crossover TSS switching are detected at adjacent time points (357 of 9767 events). However, more than a quarter of the changes at adjacent time points occur between E12-E13 (93 out of 357). This time period coincides with key developmental events such as cell specification, cell proliferation of granule cell precursors in the rhombic lip, as well as the initiation of cells migrating toward the anterior end of the cerebellum [36].

### Alternative TSS as post-transcriptional control during cerebellar development

Alternative TSSs can produce distinct mRNA isoforms that have different RNA stability and translational efficiency of the mRNA isoforms. For example, Vascular Endothelial Growth Factor A (VEGF-A) mRNA stability is regulated through alternative initiation codons that are generated through usage of alternative promoters [37]. We found that two alternative forms of Anp32a are dominantly expressed at different developmental stages in the cerebellum. The long form has 12 additional amino acids on the N-terminus compared to the short form. This difference could alter ANP32A protein stability and distribution. The role of Anp32a during cerebellar development is not known, but it is found to be involved in a variety of cellular processes in both nucleus and cytoplasm, including signaling, apoptosis, protein degradation, and morphogenesis [38]. Moreover, Anp32a is known to be a key component of the inhibitor of acetyltransferase (INHAT) complex in the nucleus, involved in regulating chromatin remodeling or transcription initiation [39]. There are suggestions that Anp32a may play important roles in the brain as the level of Anp32a is increased in Alzheimer’s disease and may be involved in the regulatory mechanism of affecting Tau phosphorylation and impairing the microtubule network and neurite outgrowth [40].

Alternative TSSs can also be a means of producing mRNA isoforms with various mRNA stability and translation efficiency. In the case of Gpc6, we found that its two forms only differ in mRNA sequence that could affect its mRNA stability and translation efficiency. Gpc6 is most abundantly expressed in the ovary, liver, and kidney, with low level expression in the nervous system [41]. In mice, Gpc6 is critical to modulating the response of the growth plate to thyroid hormones [42]; while in human, mutations in the region where Gpc6 resides on Chromosome 13 are associated with defects in endochondral ossification and cause recessive omodysplasia [43].

### Functional importance of alternative TSS during cerebellar development

Alternative TSSs can produce protein isoforms with distinct N-termini; this in turn would lead to alterations in protein function. An example would be the secreted and membrane-bound isoforms of mammalian Fos-responsive gene, Fit-1, that are generated and regulated by a pair of alternative promoters [44]. We found that during cerebellar development, the short form of Cntnap2 loses most of the functional domains present in the long form – with only the last laminin G domain retained. Cntnap2 has been found to play a role in the local differentiation of the axon into distinct functional subdomains [45]. The function of Cntnap2 short form during cerebellar development is still to be investigated, but the lack of most functional domains suggest its role as a transcriptional suppressor – through mechanism such as non-sense mediated decay [46]; or a functional competitor for the same domain binding region [47], for Cntnap2 long form counterpart during early development. During postnatal development, the short form of Cntnap2 ceases to be expressed and the long (and presumably fully functional) form is maintained at a steady level. Cntnap2 is strongly associated with autism spectrum disorders, shown in previous studies [48–50]. A knockout mouse for Cntnap2 targeted the gene’s first exon and completely eliminated the expression of the long form [51], which caused abnormalities in body size, neuronal migration and activity, and behaviour. Thus the knockout has been used as an animal model for autism [52, 53]. However, the short form of Cntnap2 should be present in the knockout, and no attention has been directed to the expression of the short form in the knockout. A mutation targeted to the C-terminus would be required to reveal Cntnap2’s overall function in considering both its long and short protein isoforms.

## Conclusion

We analyzed the cerebellar developmental time course data from the FANTOM5 project and identified 9767 TSS switching events with temporally specific dominant promoters. This is the first study to investigate the prevalence of alternative TSS usage during cerebellar development and their potential roles in transcriptional, post-transcriptional and functional regulation.
