# Correction to: Relatively frequent switching of transcription start sites during cerebellar development

**DOI:** 10.1186/s12864-017-4291-4

**Published:** 2018-01-11

**Authors:** Peter Zhang, Emmanuel Dimont, Thomas Ha, Douglas J. Swanson, Masayoshi Itoh, Hideya Kawaji, Timo Lassmann, Carsten O. Daub, Erik Arner, the FANTOM Consortium, Piero Carninci, Yoshihide Hayashizaki, Alistair R. R. Forrest, Winston Hide, Dan Goldowitz

**Affiliations:** 1 Centre for Molecular Medicine and Therapeutics, Child and Family Research Institute, Department of Medical Genetics, University of British Columbia, 950 West 28th Avenue, Vancouver, BC V5Z 4H4 Canada; 2 Department of Biostatistics, Harvard School of Public Health, 655 Huntington Ave, Boston, MA 02115 USA; 3 Harvard Stem Cell Institute, 1350 Massachusetts Ave, Cambridge, MA 02138 USA; 4 Division of Genomic Technologies, RIKEN Omics Science Center, Yokohama, Japan; 5 RIKEN Center for Life Science Technologies, Yokohama, Japan; 6 RIKEN Preventive Medicine and Diagnosis Innovation Program, Wako, Japan; 7 Telethon Kids Institute, The University of Western Australia, 100 Roberts Road, Subiaco, Subiaco, WA 6008 Australia; 8 Department of Neuroscience, Sheffield Institute of Translational Neuroscience, University of Sheffield, Room B37 385a Glossop Road, Sheffield, South Yorkshire S10 2HQ UK

## Correction

The authors of the original article [1] would like to recognize the critical contribution of core members of the FANTOM5 Consortium, who played the critical role of HeliScopeCAGE sequencing experiments, quality control of tag reads and processing of the raw sequencing data.

The revised author list includes authors Masayoshi Itoh, Hideya Kawaji, Timo Lassmann, Carsten O. Daub, Erik Arner, Piero Carninci, Yoshihide Hayashizaki and Alistair R. R. Forrest, as well as their affiliations.

The following updates to Author contributions’, Acknowledgements and Funding have also been included below:

## New Author Contributions statement

PZ, TH, DS and DG generated samples for the time series. MI and PC generated the CAGE libraries. YH, COD, ARRF and PC managed and organized the FANTOM5 project. ED, TL, HK, EA and PZ performed data analysis. PZ performed biological validation experiments. PZ, DG, ED and WH wrote the manuscript. The authors read and approved the final manuscript.

## Acknowledgements

We thank J. Yeung, J. Cairns, S. Tremblay, A. Poon, J. Wilking for support and suggestions on experimental design and manuscript preparation. We thank F. Lucero Villegas for animal management. We thank M. Larouche, D. Rains and J. Boyle for technical support. We thank Dora Pak and Anita Sham for management support and Miroslav Hatas for systems support. We would like to thank all members of the FANTOM5 consortium for contributing to generation of samples and analysis of the data-set and thank GeNAS for data production. We thank GenomeBC, National Institutes of Health, Natural Sciences and Engineering Research Council of Canada, NeuroDevNet, MEXT Japan and University of British Columbia for funding support.

## Funding

The efforts of PZ, TH, DS and DG were supported by GenomeBC and National Institutes of Health, Natural Sciences and Engineering Research Council of Canada. FANTOM5 was made possible by a Research Grant for RIKEN Omics Science Center from MEXT to YH and a grant of the Innovative Cell Biology by Innovative Technology (Cell Innovation Program) from the MEXT, Japan to YH. It was also supported by Research Grants for RIKEN Preventive Medicine and Diagnosis Innovation Program to YH and RIKEN Centre for Life Science Technologies, Division of Genomic Technologies (from the MEXT, Japan).
